# Bivalent Associations in *Mus domesticus*$2n=40$ Spermatocytes. Are They Random?

**DOI:** 10.1007/s11538-014-9992-0

**Published:** 2014-07-18

**Authors:** Julio López-Fenner, Soledad Berríos, Catalina Manieu, Jesús Page, Raúl Fernández-Donoso

**Affiliations:** 1 Departamento de Ingeniería Matemática, Universidad de La Frontera, Casilla 54-D, Temuco, Chile; 2 Programa Genética Humana, ICBM, Facultad de Medicina, Universidad de Chile, Santiago, Chile; 3 Unidad de Biología Celular, Departamento de Biología, Universidad Autónoma de Madrid, Madrid, Spain

**Keywords:** Chromosome associations in meiotic cells, Probability distributions on regular graphs, 92C37 (Cell biology), 05A17 (Partitions of sets and integers), 92-08 (Computational methods)

## Abstract

The establishment of associations between bivalents from *Mus domesticus* $2n=40$ spermatocytes is a common phenomenon that shows up during the first prophase of meiotic nuclei. In each nucleus, a seemingly random display of variable size clusters of bivalents in association is observed. These associations originate a particular nuclear architecture and determine the probability of encounters between chromosome domains. Hence, the type of randomness in associations between bivalents has nontrivial consequences. We explore different models for randomness and the associated bivalent probability distributions and find that a simple model based on randomly coloring a subset of vertices of a 6-regular graph provides best agreement with microspreads observations. The notion of randomness is thereby explained in conjunction with the underlying local geometry of the nuclear envelope.

## Introduction: The Biology

Meiosis is a cell process that occurs exclusively in germ cells of sexually reproducing organisms. At the end of this processes, haploid cells or gametes are produced. During meiotic prophase each pair of homologous chromosomes synapse forming structural units called bivalents. Phenomena like Genetic Recombination, which produce genetic variation in gametes, occur while the chromosomes are thus organized (Cobb and Handel 1998; Cohen et al. 2006; Creighton and McClintock 1931; Page and Hawley 2003; Yang and Wang 2009).

In male meiotic cells of *Mus domesticus* $2n=40$, 19 telocentric autosomal bivalents plus the XY bivalent are formed. These autosomal bivalents are very similar in morphology presenting all of them abundant pericentromeric heterochromatin towards one of the two extremes (Pardue and Gall 1970; Redi et al. 2001). The proximal telomere, the centromere, and the pericentromeric heterochromatin all together are considered to build a morphological unit, and has been named Centromere–Telomere-Complex (CTC) (Berríos et al. 2010). These CTC’s in *Mus Domesticus* $2n=40$ are attached to the nuclear envelope, and may be observed either isolated or in clusters by proximity: In early prophase cells the CTC’s of all bivalents are close together forming a unique cluster. As the meiotic prophase progresses, the nuclear volume increases and the bivalents disaggregate splitting the original cluster into a number of smaller ones. In each nucleus, a seemingly random display of variable size clusters of CTC’s is observed (Berríos et al. 2010). In this article we explore different meanings for the word randomness and the associated bivalent probability distributions.

Clearly, the associations between chromatins from different bivalents originate a particular nuclear architecture for each species, and hence determine the probability of encounters between genes for their expression or recombination (Berríos et al. 2014; Henikoff 1997; Martinez-Perez and Colaiácovo 2009). Therefore, the type of randomness in localization and associations between bivalents has nontrivial consequences.

**Fig. 1:**
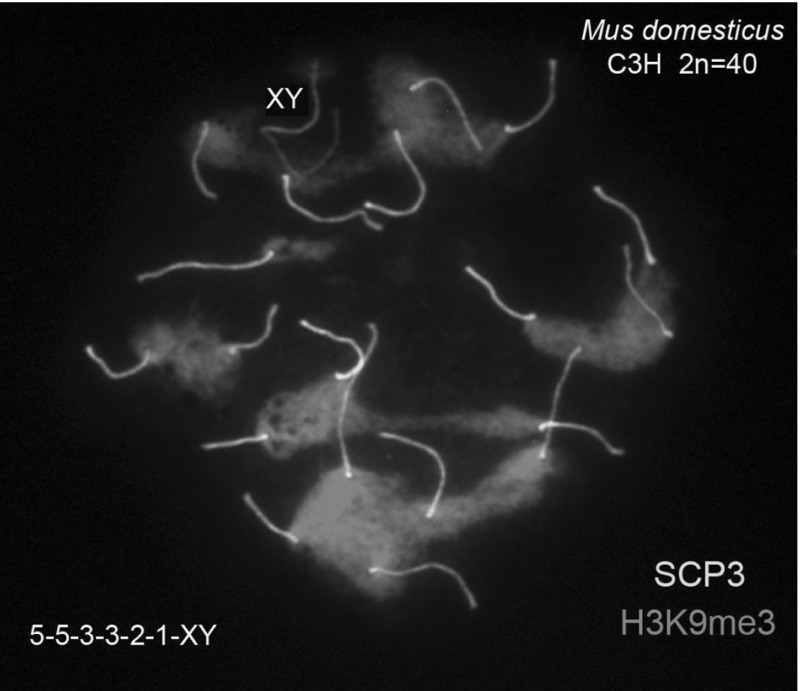
Combinations of single and associated CTC’s in spermatocyte nuclear spreads of *Mus domesticus* $2n=40$. Clusters are preserved through the nuclear envelope breakdown. Bivalents were labeled with FITC anti-SYCP3 antibodies (*green*) and the CTC’s with Texas red anti-H3K9me3 antibodies (*red*). This spermatocyte is partitioned into association clusters of size 5, 5, 3, 3, 2, 1 plus the XY. The XY bivalent does not associate with any of the 19 autosomal bivalents and was not considered in the development of the probabilistic model (Color figure online)

Notice that CTC’s associations are very strong; they are preserved despite the technical breakdown of the nuclear envelope caused in order to obtain the microspreads. In those nuclear spreads, see for example Fig. 1, the numeric combination of single and associated CTC’s per nucleus can be assessed.

The aggregation of bivalents into association clusters, labeled by their size, induces naturally a configuration space in which every partition of the number of autosomal bivalents appear as a possible candidate to be observed under microscopy. Are they random? Figure 1 shows one example of the labeling of the spermatocyte nucleous as the partition $N=19=5+5+3+3+2+1$. Hence, a first approach to randomness would be to compute the frequencies of all partitions found in observed spermatocyte nuclei.

If we denote by $\Pi (N)$ the number of partitions of $N$, it turns out that for $N=19$ there are $\Pi (19)=490$ different partitions for *Mus Domesticus* $2n=40$ spermatocytes. Establishing the association probabilities via the observed frequencies is a procedure that requires data from a number of samples that exceed largely the data obtained from inspection of microspreads.

In order to reduce the number of observations needed for quantification of the association probabilities, in Berríos et al. (2010) the notion of “*spermatocytes classes*” was introduced, according to which the class of a given spermatocyte corresponds to the size of the biggest chromocentre fragment observed. In terms of our notation, the class of a partition $N=n_1+n_2+\cdots +n_r$ would be $n_r$, if $n_1 \le n_2 \le \cdots \le n_r$.

In our first Laplace model, a scenario in which all partitions of the number $N=19$ were equally probable was considered. Here, the probability of a given class is, therefore, proportional to the number of partitions it contains (Laplace mechanism). If we denote by $\Pi (N)$ the number of partitions of $N$, and by $\Pi (N,k)$ the number of partitions of $N$ classified as belonging to class $n_r$, then the probability equals

1 $$\begin{aligned} P(n_r) = \frac{\Pi (N;n_r)}{\Pi (N)}. \end{aligned}$$

We used an algorithm in the Python programming language, described in http://code.activestate.com/recipes/218332/ and obtained the results reported in Table 1, column of Laplace figures, which we quote from Berríos et al. (2010).

**Table 1:** Comparison between predicted and observed frequencies

Classes	Laplace	Bell’s numbers	6-regular 98- cell graph	Observed
1	0.20	$\sim 10^{-11}$	0	0
2	1.84	0.08	0.89	0.5
3	6.12	11.70	13.69	11.25
4	11.02	40.33	26.26	23.25
5	14.29	30.86	23.32	23.50
6	14.50	12.31	15.50	18.50
7	13.27	3.62	9.26	10.25
8	10.61	0.88	5.27	6.50
9	8.37	0.18	2.91	4.25
10	6.12	0.03	1.52	1.25
11	4.49	$\sim$10$^{-3}$	0.77	0.75
12	3.06	$\sim$10$^{-4}$	0.37	0
13	2.24	$\sim$10$^{-5}$	0.16	0
14	1.43	$\sim$10$^{-5}$	0.06	0
15	1.02	$\sim$10$^{-6}$	0.02	0
16	0.61	$\sim$10$^{-8}$	0.01	0
17	0.41	$\sim$10$^{-9}$	0	0
18	0.20	$\sim$10$^{-10}$	0	0
19	0.20	$\sim$10$^{-11}$	0	0
Total	100.0	100.0	100.0	100.0

Laplace figures quoted from Berríos et al. (2010)

Our second approach consisted in selecting partitions from an abstract set $\Omega$ with $|\Omega |=N$ elements. $\Omega$ represents a generic *Mus domesticus* $2n=40$ spermatocyte, the elements are the 19 bivalents. Hence, choosing at random a subset of $\Omega$ corresponds naturally to a random selection of interacting bivalents of the spermatocyte. How many selections are there? It is well known that this number is exactly $B_N$, the $N$-th Bell Number (see Berend and Tassa (2010) or Rota (1964) for details).

If all partitions of $\Omega$ are equally probable (Laplace mechanism again), then as in Eq. (1) the probability of a given partition is

2 $$\begin{aligned} P(n_r) = \frac{\Pi (N;n_r)}{B_N} \end{aligned}$$

with the difference that now

3 $$\begin{aligned} \Pi (N;n_r) = \sum _{N=n_1+n_2+\cdots +n_r} \pi [n_1,n_2,\ldots n_r], \end{aligned}$$

where

4 $$\begin{aligned} \pi [n_1,n_2,\ldots ,n_r] = \frac{N!}{(m_1!)^{k_1}(m_2!)^{k_2}\cdots (m_p!)^{k_p}}\cdot \frac{1}{k_1!k_2! \cdot k_p!}. \end{aligned}$$

The relationship between the $n$’s, $m$’s and $k$’s is the following: We need to take into account the multiplicities of the $n_i$’s, $i=1,2,\ldots r$: $N=\sum _{i=1}^r n_i = \sum _{j=1}^p k_j m_j$ such that $\forall m_j\; \exists n_i$ such that $m_j=n_i$ and $1 < m_j < m_{j+1} \le N$, and call $k_j$ the “multiplicity” of the subcluster $m_j=n_i$. In other words, $k_j$ represents the number of clusters with the same cardinality $n_i$. Thus, the sum at the right includes the multiplicities of the single $n_i$’s. For example, partition $5=1+1+1+2$ is written in a unique fashion as $5 = 3\cdot 1 + 1\cdot 2$, etc.

Table 1 shows the results for $N=19$, Bell’s number’s column.

## Geometric Probabilities

In both previous approaches, the underlying Laplace assumption of equal probability for the partitions assign different probabilities to the outcomes of each measurement (cluster determination for a given spermatocyte). With them, we can compute the probability for each class and contrast with available data. We explore a third approach, obtained from the inclusion of geometric properties of the configuration space, a procedure known as “the geometric probability approach” Prokhorov (2014). As described in Berríos et al. (2010), the cluster formation (disaggregation of the prophase bouquet with CTC’s attached to the nuclear envelope) can be modeled as a placement of single CTC’s upon the inner surface of the nuclear envelope by means of a Laplace mechanism. One local representation of such a structure consists in a 2-D surface with hexagonal tiling (see Berríos et al. (2010) for a complete description). Each cell from this tessellation allows for exactly six neighbors. While this description fits the local representation of the inner side of the nuclear membrane, it is also a known fact that there can be no tiling of the surface of a sphere with regular hexagonal cells. (Equivalently, a 2-D surface made of hexagonal cells cannot be folded into a sphere).

One way to circumvent this problem consists in introducing an hexagonal tessellation on a torus (or toroidal 2-D surface) which appears when a 2-D rectangle is folded such that the upper and lower edges are adjacent, and the left and right edges also.

This implementation furnishes a geometric realization of a coloring problem in graphs: Let $G=(V,E)$ be a 6-regular graph with $|V|=2n^2$ vertices. Recall that in an $r$- regular graph all vertices have degree $r$, $\text {deg}(v)=r$, $\forall v \in V$. Now mark (color) $N=19$ vertices “at random” and determine the induced partition of $N$ in clusters $N=n_1+n_2+\cdots +n_r$ in which each cluster is a connected subset of colored vertices. By repeating, frequencies of induced clusterings can be obtained.

Simulations were implemented in Python Programming Language version 2.7 (Python Programming Language 2014) on an Intel(R) Core(TM)2 Quad CPU Q9400 @ 2.66 GHz machine with Linux-Mint version 2.6.38-8.

**Fig. 2:**
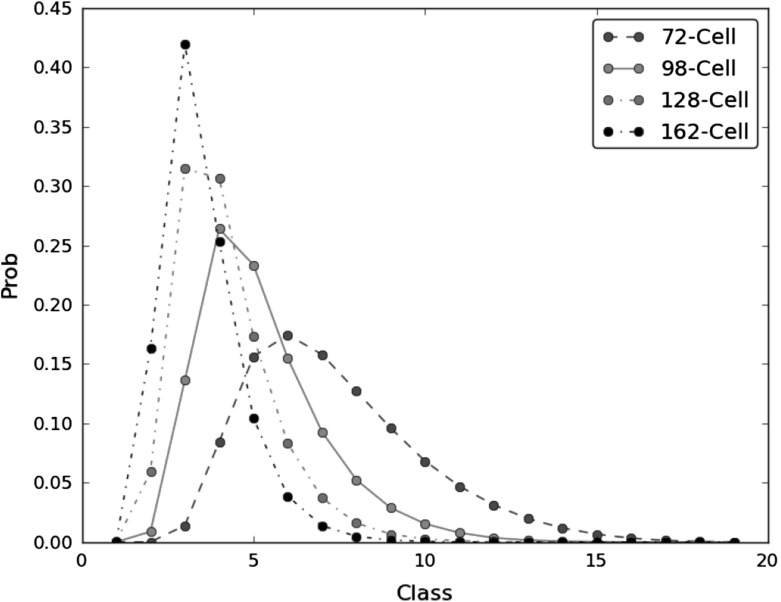
Graphical representation of simulated frequencies for classes (Table 1). Depicted are the results obtained for a 6-regular graph with 72, 98, 128, and 162 vertices, respectively, after $10^6$ iterations (Color figure online)

We computed the frequencies of all partitions (clusters) obtained by random coloring $N=19$ vertices on a 6-regular graph with $|V|=M=2 n^2$ vertices and $n= 6, 7, 8$, and 9, respectively, i.e., 6-regular graphs with 72, 98, 128, and 162 vertices (or cells). This procedure was repeated NITER $= 10^6$ times, yielding computed frequencies with 2 significative figures. Results are presented in Table 2 (Fig. 2).

As expected from first principles, with increasing number $n$, i.e., with increasing number of vertices in the 6-regular graph, the partitions tend to be disaggregated, i.e., lower classes become more probable.

**Table 2:** Simulated frequencies of spermatocytes per class in tori with different number of cells

Classes	72-Cell	98-Cell	128-Cell	162-Cell
1	0.0000	0.0001	0.0021	0.0388
2	0.0010	0.8854	0.5921	16.3799
3	1.3907	13.6858	31.5344	41.8403
4	8.3891	26.2594	30.5366	25.3575
5	15.6196	23.3200	17.3059	10.4571
6	17.5211	15.4996	8.2455	3.8606
7	15.7197	9.2585	3.7015	1.3752
8	12.7534	5.2693	1.6303	0.4753
9	9.5921	2.9055	0.6843	0.1526
10	6.8161	1.5222	0.2758	0.0443
11	4.7241	0.7692	0.1043	0.0140
12	3.1112	0.3725	0.0366	0.0036
13	1.9779	0.1589	0.0101	0.0006
14	1.1957	0.0611	0.0026	0.0002
15	0.6576	0.0246	0.0007	0.0000
16	0.3302	0.0067	0.0002	0.0000
17	0.1408	0.0006	0.0000	0.0000
18	0.0434	0.0005	0.0000	0.0000
19	0.0073	0.0001	0.0000	0.0000
Total	100.0	100.0	100.0	100.0

**Fig. 3:**
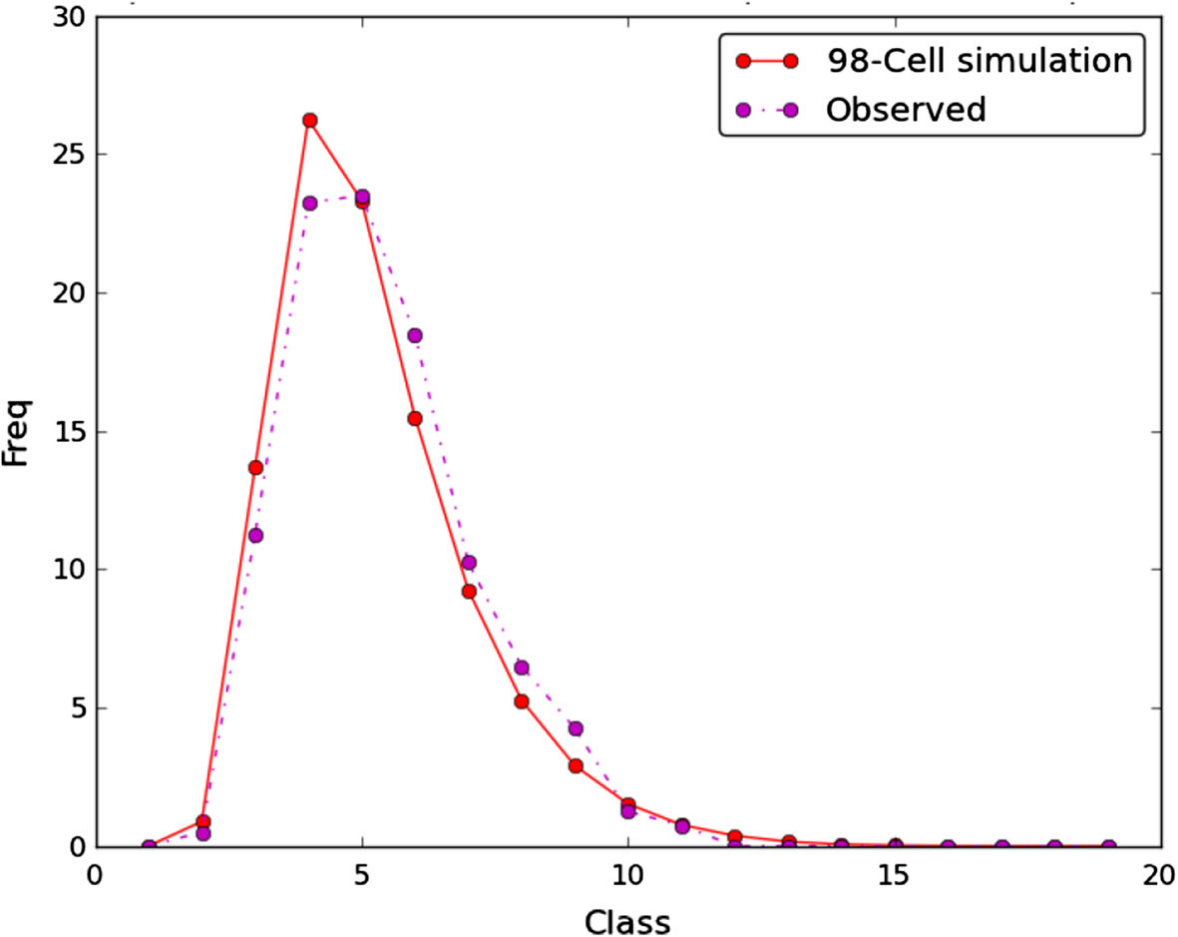
Observed versus simulated frequencies: comparison made from observed frequencies of CTC Classes, determined in microspreads from pachytene spermatocytes of *Mus domesticus* reported in Berríos et al. (2010), and the 98-Cell simulation after $10^6$ iterations (Color figure online)

Comparison with the observed frequencies of CTC Classes as determined in microspreads from pachytene spermatocytes of *Mus domesticus* $2n=40$Berríos et al. (2010) shows that best agreement with computed (simulated) frequencies is achieved with the $|V|=98$ configuration (Fig. 3). Since cells of the tessellation are the places for locating the CTC’s, and each CTC has a definite size of approximately $1 \upmu$m in diameter, this number can be back interpreted as the size (or proportion) of the effective surface of the nuclear envelope in which uniform random chromosome association appears to be equivalent to a disagregation process of the bouquet from earlier stages of the meiotic prophase.

**Fig. 4:**
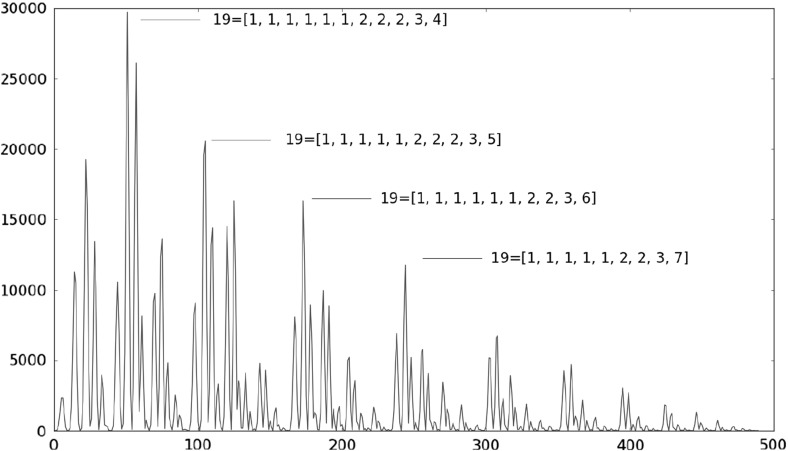
Simulated frequencies of all partitions of $N = 19$ after $10^6$ *iterations in 98 cell torus*: some maximum probability partitions are pointed out

Figure 4 shows the simulated frequencies of each one of the 490 partitions of $N=19$. Recall that partitions are indexed in growing form, thus partition number 1 corresponds to $19= 1+1+\cdots +1$, etc., until partition number 490, which is $19=19$.

Notice that contrary to our assumption made in Berríos et al. (2010), partitions in this model are far from equiprobable. The geometrical nature of the association space (the 6-regular graph visualized as an hexagonally tessellated torus) seems to play a rôle in the quasi fractal form of the frequencies (in the sense that inside each class a distribution similar to the class-distribution is found), yet it lies beyond the scope of this article to explore this finding.

A different approach, which we did not pursue in this article, would have been to consider the complete graph $K_{19}$, in which all vertices are connected, and then extract randomly a subset of edges, thereby determining the resulting partition in connected components. While this approach (size distribution of connected components in randomly induced subgraphs) has been considered elsewhere, see for example Reydis et al. (1997), we did not follow it because on one side for $K_N$, which has $N(N-1)/2$ edges exactly, the complete disconnected partition $N=1+1+\cdots +1$ would be obtained only after removing all edges. On the other side, the procedure would not reflect the observed fact that the associated clustering is preserved through the prophase or even the first meiotic metaphase. We will come to this subject in a forthcoming article.

From the results obtained so far, we found that already the 6-regular graph with 98 vertices fitted best the observations with a $\chi ^2$ value of 1.9169 and *p* value of 0.9272. See Table 1, column 6-regular 98-cell graph and compare with observed data. As a further check, we restricted the universe of observations by considering a subsample of 93 spermatocytes of *Mus domesticus* $2n=40$, all of them adscribed to class 4. The class $n_r=4$ contains 54 different partitions, ranging from $19=1+1+\cdots +1 +4$, until $19=3+4+4+4+4$. In the sample, only 28 different partitions were identified. Since the universe has been restricted to be of class 4, direct comparison with the frequencies computed earlier is not directly possible, unless conditional frequencies are considered, as usual. These frequencies are reported in Table 3.

**Table 3:** Observed frequencies (OF) and simulated conditional frequencies (SCF) for class 4 partitions of $N=19$

Partition	OF	SCF
$1+\cdots +1 + 2 + 2 + 2 +4$	7.5	2.8
$1+\cdots +1 + 2+2+2+2+4$	1.1	4.1
$1+\cdots +1+ 2+2+2+2+2+4$	2.2	2.7
$1+\cdots + 1 +2 +3 +4$	4.3	2.2
$1+\cdots + 1 +2+2 +3 +4$	6.5	8.0
$1+\cdots + 1 +2 +2+2+3 +4$	3.2	11.6
$1+ 1 +1+1+2 +2+2+2+3 +4$	6.5	6.6
$1+\cdots +1 +3 +3 +4$	1.1	1.1
$1+\cdots + 1 +2 +3 +3 +4$	4.3	6.4
$1+1+1+1+ 1 +2+2+3 +3 +4$	7.5	10.1
$1+1 + 1 +2 +2+2+3+3 +4$	4.3	4.9
$1+1+ 1+1+1+1 +3+3 +3 +4$	4.3	1.4
$1+1+1 + 1 +2+3+3 +3 +4$	3.2	3.1
$1 + 1 +2+2+3+3 +3 +4$	2.2	1.3
$1+1 + 1 +3+3 +3 +3+4$	3.2	0.3
$1+\cdots + 1+ 2 + 4 + 4$	1.1	1.3
$1+\cdots + 1+ 2 + 2+ 4 + 4$	4.3	3.5
$1+\cdots + 1+ 2 +2+2+ 4 + 4$	3.2	3.7
$1+1 + 1+ 2+2+2+2 + 4 + 4$	2.2	1.4
$1+\cdots + 1 +2 +3+4 +4$	4.3	4.8
$1+1+1 + 1 +2+2+ 3+4 +4$	7.5	5.2
$1 + 1 +2+2+2 +3 +4+4$	4.3	1.5
$1+\cdots + 1 +3+3 +4 +4$	3.2	1.3
$1+1 + 1 +2 +3 +3+4+4$	2.2	1.9
$1+1+1 + 1+ 2 + 4 +4 + 4$	3.2	1.0
$1+1+1 + 1+ 3+ 4 + 4+4$	1.1	0.4
$1 + 1+ 2 +3+4+ 4 + 4$	1.1	0.4
$1+3+3+4 + 4 + 4$	1.1	0.0
Total	100.0	100.0

The observed frequencies (OF) were obtained from the associated partitions recorded in 93 Class 4 *Mus domesticus* $2n=40$ spermatocytes. Simulated conditional frequencies (SCF) correspond to the ratio between simulated frequencies of the corresponding partition and the total simulated frequency for class 4 ($\sim$26 %), times 100

We notice a striking similarity for the behavior of observed and simulated conditional frequencies overall, save for partition 19 = 1 + 1 + 1 + 1 + 1 + 1 + 2 + 2 + 2 + 3 + 4, that was only observed in 3 out of 93 spermatocytes, while in our simulations, it has the highest probability of occurence (about 30 %) with conditional frequency of 11.6 % inside class 4.

## Discussion

Understanding the topologic organization and interactions between chromosome domains inside nuclei, in which gene expression takes place, requires—among other conditions—the compilation of a significant set of observational data as well as the design of mathematical models, like those developed for somatic nuclei (Amrichová et al. 2003; Cerda et al. 1999; Sanyal et al. 2011). Meiotic Prophase-I cells offer some advantages for this type of analysis, because they exhibit functional nuclei in which bivalents can be neatly distinguished, as well as their interactions. Moreover, *Mus domesticus* $2n=40$ spermatocytes exhibit 19 autosomal bivalents, all of them similar in morphology and with plenty of surrounding heterochromatin at their proximal ends, so that their meiotic prophase nuclei constitute an excellent scenario for studying both the nuclear topology and the associations between their bivalents through pericentromeric heterochromatic domains. The XY-bivalent was not considered because it does not associate with any of the 19 autosomal bivalents.

The model in Berríos et al. (2010) started from two main assumptions, namely the indistinguishability between the $N=19$ CTC’s and that all partitions of the number $N=19$ were equiprobable. A further hypothesis was the condition that once placed randomly upon the nuclear envelope, each CTC admitted six potential neighbors. As stated earlier in this article, we maintained the hypothesis of equal number of neighbors and the implicit local symmetry of the surface of the nuclear envelope under rotations.

Therefore, we changed the assumption of a flat surface with hexagonal tiling to a torus that tessellated hexagonally, by defining the opposite edges of the 2-D surface to be adjacent. This procedure actually build a realization of a 6-regular graph in which vertices are the available cells for the CTC’s to take place. Hence, an algorithm for random coloring of the $N=19$ CTC’s (vertices) allow for computer simulation of the frequencies of occurrence of each partition.

Contrary to the assumption made in Berríos et al. (2010), we found that the model using graph coloring in 6-regular graphs exhibit closer frequencies to those observed than in the other formulations: Under this model partitions are far from equiprobable yet perfectly random.

In this sense, we found that a 6-regular graph of 98 vertices visualized as a torus with 98 hexagonal cells, appear as the best configuration in terms of consistency with the observed data. Since CTC’s at the base of the envelope measure approximately 1 $\upmu$m$^2$, we could propose that the proportion of the envelope involved in the random process of associations between bivalents takes about one sixth of the total envelope (measuring approximately 620 $\upmu$m$^2$). Thus, we do not only have gained a probabilistic description for the association between bivalents, but also an estimation of the size of the nuclear envelope in which these associations could take place in a random fashion.

As for the simulated frequencies themselves, the distribution of probabilities inside each class exhibit a form in which some partitions appear to be more probable than others. This phenomenon is also consistent with the observations, as discussed in the previous section for class 4.

It is worth noticing that there are other simple geometric models that could be used for establishing a notion of randomness for the observed associations. For example, in Carlton and Cande (2002), randomly selected points from a sphere are considered. We decided not to follow this approach because in this model, alone the definition of the induced clustering of association would exceed the complexity of the description we were seeking for. Instead, the approach described in this work combines simplicity of the model with adequacy of fitting the data. Once the individual probabilities of the clusters are determined, the model can be used for answering other questions. For example, assume a given pair of CTC’s can be selected by differential staining. The question can be posed about the probability that these two bivalents appear in association in the same cluster, or the probability that they are not associated, nor among them, nor among other. By simple probability computations, the answer will be that in approximately 12 % of the observed microspreads, any given pair of bivalents would belong to the same cluster and that in approximately 88 % they would be found in different clusters.

These findings induce us to propose that the biological phenomenon of association among bivalents during pachytene may be appropriately described under a notion of pure random association in a suitable space of configurations like the one discussed here. This approach may provide some light of theoretical nature upon the dynamics of chromosomes and their possible interactions, which ultimately could be a key to a better understanding of the genome’s functional organization.
